# Supplementary material for: Time trends in and factors associated with repeat adolescent birth in Uganda: Analysis of six demographic and health surveys
Source: PLoS One. 2020 Apr 14;15(4):e0231557. doi: 10.1371/journal.pone.0231557 (PMC7156070; doi:10.1371/journal.pone.0231557)
Supplement: S1 Table — (PDF) [file pone.0231557.s001.pdf]

S1 table: Percent point difference in repeat adolescent birth between surveys among Uganda women age 20-24 years,all UDHS surveys

ALL WOMEN AGE 20-24 YEARS				
**Intervals**	**5 years**	**5 years**	**5 years**	**5 years**
	**1988/89-1995**	**1995-2000/01**	**2000/01-2006**	**2006-2011**
**Percentage Point difference (P-value)**	-0.3 (0.864)	+2.3 (0.144)	-3.1 (0.043)	-3.1 (0.031)
**Intervals**	**5 years**	**First 15 years**	**Last 15 years**	**30 years**
	**2011-2016**	**1988/89-2000**	**2006-2016**	**1988/89-2016**
**Percentage Point difference (P-value)**	-4.6 (<0.001)	+2.0 (<0.001)	-7.7 (<0.001)	-8.8 (<0.001)
**WOMEN 20-24 YEARS WITH 1ST BIRTH <18 YEARS**				
**Intervals**	**5 years**	**5 years**	**5 years**	**5 years**
	**1988/89-1995**	**1995-2000/01**	**2000/01-2006**	**2006-2011**
**Percentage Point difference (P-value)**	+3.3 (0.289)	+1.1 (0.689)	+3.5 (0.197)	-5.1 (0.073)
**Intervals**	**5 years**	**First 15 years**	**Last 15 years**	**30 years**
	**2011-2016**	**1988/89-2000**	**2006-2016**	**1988/89-2016**
**Percentage point difference (P-value)**	-6.1 (0.019)	+4.4 (0.154)	-11.2 (<0.001)	-3.3 (0.251)
